# The COVID-19 pandemic impact on prenatal depression : A Cross-sectional comparative study

**DOI:** 10.1192/j.eurpsy.2022.1290

**Published:** 2022-09-01

**Authors:** O. Maatouk, E. Khelifa, K. Nourchene, B. Abassi, I. Bouguerra, F. Amdouni, A. Ben Amor, L. Mnif

**Affiliations:** 1 Razi Hospital, F Adult Psychiatry Department, Manouba, Tunisia; 2 Razi Hospital, Psychiatry Ibn Omran, Manouba, Tunisia; 3 Errazi hospital-Mannouba , F, Ben Arous, Tunisia

**Keywords:** Depression, Impact, pregnant, Coronavirus-2019

## Abstract

**Introduction:**

Coronavirus disease 2019 (COVID-19) is the current world issue, with huge impact on mental health. More specifically,we expect that it will have a naocif effect on the pregnant women’s mental health and their well being, since they are more likely to be hospitalized *and* require more intensive care units admission than non- *pregnant women.*

**Objectives:**

The aim of this work was to evaluate the evolution of depression symptoms in the time of pandemic and their associated factors.

**Methods:**

In the current work, we conducted a comparative in field cross-sectional study. We compared depressive scores and prevalences before and after the COVID-19 outbreak in Tunisia in pregnant women.The sampling period was outside the lockdown period to avoid quarantine bias. The sampling period was from September to October 2020.

**Results:**

showed a significantly higher prevalence of depressive symptoms in expecting mothers during the pandemic. Multivariate analysis showed that the pandemic multiplied by 3 the risk of severe depression symptoms. The impact of the COVID-19 period on depression was independent of sociodemographic and obstetric changes related to the pandemic.

**Conclusions:**

These results highlighted the emergency of preparing strategies to avoid post-partum psychiatric disorders and to enable a healthy development of born. Screening the post-partum depression and assessing the mother-children early interactions should be considered in the up-coming births.

**Disclosure:**

No significant relationships.

